# Evaluating the Potential of Metaverse to Elicit Therapy-Related Emotions Among Individuals With Depression: Controlled Pilot Study of Cognitive and Emotional Responses

**DOI:** 10.2196/82422

**Published:** 2025-12-22

**Authors:** Fabiha Islam, Purushothaman Muthukanagaraj, Mei-Hsiu Chen, Liang Zhan, Alex Leow, Chao Shi

**Affiliations:** 1 School of Systems Science and Industrial Engineering, Binghamton University, Engineering Building – Room R20, 4400 Vestal Parkway East, Binghamton, NY, 13902, United States, 1-607-777-5018; 2 Department of Psychiatry, UHS Binghamton General Hospital, Binghamton, NY, United States; 3 Department of Mathematics and Statistics, Binghamton University, Binghamton, NY, United States; 4 Department of Electrical and Computer Engineering, University of Pittsburgh, Pittsburgh, PA, United States; 5 Department of Psychiatry, University of Illinois Chicago, Chicago, IL, United States

**Keywords:** depression, emotional response, mental health, metaverse, second life

## Abstract

**Background:**

Depression, a prevalent mental health condition, affects millions worldwide. Many face significant barriers to accessing effective therapy. Metaverse has become a promising platform for addressing these challenges. Its 3D virtual environments allow users to engage through customizable avatars, supporting the elicitation of therapy-related emotions, an essential component of therapeutic interventions.

**Objective:**

This pilot study investigates the potential of the Metaverse to elicit therapy-related emotional responses in individuals experiencing depression. The objectives are to assess emotional arousal, mental workload (MWL), and affective engagement during an emotional elicitation task in a Metaverse environment and compare them with those observed in a similar real-world setting.

**Methods:**

We conducted a between-subjects experiment involving 28 participants: 14 had self-reported depression symptoms; 14 had minimal or no depression. Participants were assigned to a real-world environment or a Metaverse environment. Second Life (SL), a persistent 3D virtual world that utilizes human-like avatars for navigation, was the Metaverse environment. A desktop computer represented the real-world setting. Participants watched emotion-eliciting video clips to evoke amusement, anger, disgust, fear, and sadness. They reported their emotional arousal using the 16-item self-report inventory, MWL using the NASA Task Load Index, and emotional engagement using the Positive and Negative Affect Schedule.

**Results:**

Regardless of the environment, participants with self-reported depression symptoms reported significantly higher amusement (*F*_1,24_=4.882, *P*=.04; self-reported depression: mean 5.00, SD 2.481; minimal or no depression: mean 3.07, SD 1.940), anger (*F*_1,24_=7.067, *P*=.01; self-reported depression: mean 4.79, SD 2.190; minimal or no depression: mean 2.71, SD 1.899), sadness (*F*_1,24_=4.353, *P*=.048; self-reported depression: mean 5.21, SD 2.293; minimal or no depression: mean 3.50, SD 1.912), and perceived performance (*F*_1,24_=4.939, *P*=.04; self-reported depression: mean 91.07, SD 90.302; minimal or no depression: mean 31.07, SD 36.278) than those with minimal or no depression. Participants with minimal or no depression reported lower perceived effort in the SL environment than in the real-world setting (SL: mean 40.71, SD 38.016; real world: mean 158.571, SD 120.411; *P*=.02), indicating Metaverse’s potential to alleviate cognitive stress during emotionally eliciting tasks. There were no significant differences in perceived MWL or emotional engagement between the real-world and Metaverse settings.

**Conclusions:**

Individuals with self-reported depression symptoms exhibited heightened emotional responses across environmental settings, indicating the Metaverse may potentially elicit emotional arousal comparable to that in traditional settings. Participants with minimal or no depression symptoms reported lower perceived effort in the Metaverse, suggesting that virtual environments may reduce cognitive strain during emotionally evocative tasks. The lack of significant differences in emotional arousal, overall MWL, or emotional engagement across environments tentatively suggests that the Metaverse may deliver emotional experiences like those of real-world contexts. Collectively, this provides preliminary evidence that screen-based Metaverse platforms can elicit emotional responses like those commonly engaged during therapeutic depression interventions.

## Introduction

### Depressive Disorder and the Metaverse

Depressive disorder is a widespread mental health condition characterized by persistent feelings of sadness, low mood, and reduced interest or pleasure in activities [1-3]. According to the World Health Organization (WHO), as of 2023, depressive disorders and other mental health issues affected approximately 280 million people globally. It is estimated that around 3.8% of the global population experience depression, including 5% of adults (4% of men and 6% of women) and 5.7% of adults older than 60 years [2]. Symptoms of depression include irritability, prolonged disinterest or lack of pleasure in daily activities, impaired concentration and decision-making abilities, fatigue, sleep abnormalities, fluctuations in appetite and weight, pessimism, hopelessness, persistent sadness, restlessness, and thoughts of suicide [1,2,4-8]. The obstacles to accessing mental care services include workforce shortages, limited awareness and understanding, financial burdens, social stigma, and insufficient time for mental health discussions during medical appointments [5,9]. One way to overcome those obstacles is to use digital therapeutics or digital health in psychotherapy. Digital mental health services have evolved rapidly, aiming to enhance the efficiency and quality of health care services, including diagnosis, monitoring, treatment, and data management, through digital communication technologies [10,11]. However, most current digital mental health services only allow text or voice-based communication (eg, Tess [12], Woebot Health [13], WYSA [14]), which limits their capacity to elicit the emotional arousal and engagement that visually dynamic, real-time environments like those in the Metaverse can support.

The Metaverse is a 3D environment where users can interact with other users using avatars in a virtual world. It is a broad concept that uses a variety of technologies, including virtual reality, augmented reality, mixed reality, cryptocurrency, and the internet, to facilitate social interactions and user-generated content in a persistent digital environment across numerous domains, including economics, ecology, and health care [11,15-17]. This expansive Metaverse network allows users to experience a fully immersive environment, creating the sense of being inside the internet, where they can engage with other users, objects, and digital spaces as unique avatars, replicating real-world activities like socializing, working, and playing in a virtual setting [17,18]. By integrating physical and digital environments through devices such as tablets and smartphones, the Metaverse can connect various technologies, including artificial intelligence (AI), blockchain, tangible interfaces, and the Internet of Things, within 3D environments. This technology integration enables seamless interactions between virtual and real worlds, opening new possibilities for applications across multiple sectors, including mental health services [19].

### Application of Metaverse in Mental Health

The Metaverse has recently gained attention as a promising platform for mental health interventions, offering safe, immersive experiences for therapies such as cognitive-behavioral therapy, exposure therapy, and other virtual reality–based treatments [20]. It has been applied in the treatment of depression, arachnophobia, astraphobia, ophidiophobia, post-traumatic stress disorder, anxiety, and eating disorders [16,20,21]. Individuals who feel lonely or stigmatized in their physical environment may find social support and a sense of community in the Metaverse. The controlled environment of the Metaverse allows patients to confront various stimuli causing anxiety or fear, such as public speaking, heights, or spiders [20]. Besides, users can interact with mental health professionals using customizable, anonymous avatars in simulated environments, which may fascinate individuals reluctant to seek in-person treatment [21,22]. Several studies have demonstrated the effectiveness of Metaverse-based interventions. Ezawa et al [23] found that a coach-led cognitive behavioral program delivered through the Metaverse app, Innerworld, significantly reduced depression and anxiety symptoms, with participants engaging as customizable avatars, fostering anonymity and engagement. Athar et al [24] developed a Metaverse-based AI recommendation system for patients with anxiety and depression that analyzed clinical notes and user-uploaded survey responses to predict condition severity and recommend treatments, including medications, psychiatrist referrals, and therapeutic activities. Buragohain et al [25] also found that Metaverse-based platforms such as NightWare, Freespira, EndeavorRx, and Sleepio can provide significant benefits, with clinical evidence supporting their effectiveness for treating conditions like post-traumatic stress disorder, anxiety, and attention deficit hyperactivity disorder. Beyond clinical applications, research indicates that users generally respond positively to the Metaverse. Mahmoud [15] found that people express positive reactions toward the Metaverse, with contentment, curiosity, and excitement being the most common sentiments, reflecting enthusiasm for its potential benefits in virtual environments. However, the Metaverse also brings notable risks. Within the Metaverse, individuals often feel more relaxed, which may increase their willingness to engage in deviant behaviors such as harassment, data exploitation, privacy breaches, and unregulated gambling [18] while also raising concerns about physical discomfort; emotional states such as nervousness; negative sentiment; and psychological effects, including mental fatigue and stress [15]. Despite growing interest and applications, little fundamental research has examined whether the Metaverse can elicit the range of emotions necessary for effective therapy without adversely affecting users’ emotional or psychological states. To address this gap, this study evaluated the efficacy of the Metaverse using Second Life (SL), one of the most widely used Metaverse platforms, focusing on its ability to elicit emotional responses.

### SL as a Therapeutic Platform

SL is a persistent, 3D virtual world designed to simulate various aspects of real life. Created by Linden Research Inc and released in 2003, SL offers users a virtual world where they can create and personalize their online identities [18,26]. The platform has over 9 million registered accounts, more than 7.1 million total website visits, a daily average of 42,000 to 46,000 concurrent users, and 26,716 active regions [27]. Users can navigate the SL environment through human-like avatars, which are customizable and can be animated to express body language in unique and creative ways [26,28-33]. The virtual world consists of interconnected regions, including land, water, and sky, where users can freely alter their avatars’ appearances and engage with other community members. It supports diverse modes of communication, including text messaging, voice chat, and note cards, creating a dynamic space for social engagement [32-34]. Users can also create, buy, or sell objects through the SL marketplace on the web using Linden Dollars, a virtual currency that can be exchanged for real-world money [26,28]. These features render SL a distinctive platform for creative expression and social interaction while also demonstrating substantial potential for telehealth and therapeutic applications.

SL has emerged as a promising Metaverse platform for therapeutic interventions and eliciting emotional experiences. Its virtual environments allow individuals to explore complex psychological and emotional processes in a safe, controlled setting. Research has shown that SL can support meaningful psychological and emotional experiences across diverse populations. For example, Davis and Alexanian [35] found that participants from disability-focused SL communities used child avatars to revisit past trauma, engage in nurturing interactions, and cultivate self-compassion while reducing emotional reactivity. Vanderburg et al [32] used SL to study emotional experiences, finding that its immersive and anonymous environment enabled participants to express emotions and reflect on trauma-related experiences. SL has also been applied in structured therapeutic contexts, showing benefits for relaxation and mindfulness [29], social cognition training in high-functioning autism [36], and cognitive-behavioral therapy for social anxiety disorder [33]. Additionally, SL has been reported as an engaging, interactive platform for creating dynamic, realistic scenarios. Rudolphi-Solero et al [34] found that participants in a virtual radiology competition rated SL as highly engaging, interactive, and valuable for learning and skill development. Similarly, Gout et al [28] developed an SL-based disaster simulation that enabled flexible, immersive training with realistic scenarios and physiological victim models, effectively supporting participants’ experiential learning and practical skill acquisition. Despite these applications, few studies have specifically examined SL’s effectiveness for eliciting emotional responses within therapeutic contexts. To address this gap, our study developed a dedicated SL island where participants could engage in activities using anonymous avatars that were designed to evoke emotional experiences. Considering SL’s interactive nature, it was essential to investigate how such virtual environments may impact emotional arousal and cognitive load, particularly among individuals with depression.

### Depression, Emotional Arousal, and Mental Load

Individuals with depression exhibit an abnormal physiological arousal pattern, known as hyperarousal, which is linked to emotional and cognitive responses. Emotional arousal encompasses the brain and bodily changes that occur in response to emotionally charged stimuli, serving as an indicator of how intensely a person reacts emotionally [37]. Depressed individuals also display heightened emotional reactivity, often interpreting external stimuli as more negative and stressful than nondepressed individuals [38]. This intensified sensitivity is further reinforced by repetitive negative thinking patterns, which can prolong emotional arousal and contribute to a recurring cycle of depressive symptoms [39]. Consistent with this pattern, Wenzler et al [38] observed that, when viewing negative emotion-eliciting stimuli, depressed participants rated them as more negative than healthy controls, highlighting their amplified subjective emotional responses. Additionally, recent research by Benau et al [40] indicated that individuals with depression showed increased sensitivity to emotional stimuli that are self-relevant. In contrast, their responses to non-self-relevant stimuli were reduced. This suggests that emotional reactivity in depression depends on the personal significance of the stimulus. Emotional processes are inherently multidimensional, including affective, physiological, and behavioral components, and are commonly studied through emotion-induction paradigms using pictures or film. Supporting the importance of stimulus realism in eliciting emotional responses, Berretz et al [41] found that highly realistic, specifically disgusting stimuli evoked stronger aversive emotional responses than less realistic ones, such as pictorial presentations. Depression is also associated with increased sensitivity to mental load and stress, which may prolong symptoms and impair functioning. Bowie et al [42] found that individuals with greater depression severity are more likely to disengage from cognitively demanding tasks. Similarly, Lerner et al [43] showed that stress is linked to depression and that depressed individuals exhibit impaired performance compared with non-depressed workers. Together, these findings highlight the need to better understand the emotional arousal and mental workload (MWL) of individuals with depression within Metaverse environments, informing its potential as an effective digital therapeutic intervention.

### Research Gap and Study Aim

Although various studies have assessed the potential of Metaverse environments for mental health therapy, a significant research gap remains in investigating their effectiveness for eliciting targeted emotional responses, which may affect the realism of therapy. Specifically, there is a lack of studies examining the Metaverse platform’s ability to evoke emotional reactions comparable to those experienced in real-world therapy environments. Furthermore, few studies have measured perceived MWL and emotional engagement while performing emotionally engaging tasks in Metaverse environments compared with real-world settings. This pilot study addressed this gap by evaluating the potential of a Metaverse environment to elicit emotions relevant to therapy and by investigating whether depression severity influences these responses. The objectives of this study were to assess individuals’ emotional arousal, perception of MWL, and emotional engagement while performing an emotional elicitation task in a Metaverse setting or a real-world setting and to compare these measures between the two environments to determine the effectiveness of the Metaverse at eliciting emotions.

## Methods

### Study Design

In this study, we conducted a controlled experiment to evaluate the effectiveness of a Metaverse environment for eliciting emotional responses compared with a real-world setting. Participants were categorized as having minimal or no depression symptoms or self-reported depression symptoms based on their self-reported scores on the Patient Health Questionnaire-9 (PHQ-9) [44]. We utilized a 2 (environment: Metaverse and real world) × 2 (depression severity: minimal or no depression symptoms and self-reported depression) factorial design to assess the main and interaction effects of environment and depression severity on emotional arousal, perception of MWL, and emotional engagement while participants performed an emotional-eliciting task.

### Real-World Settings

**Figure 1.**
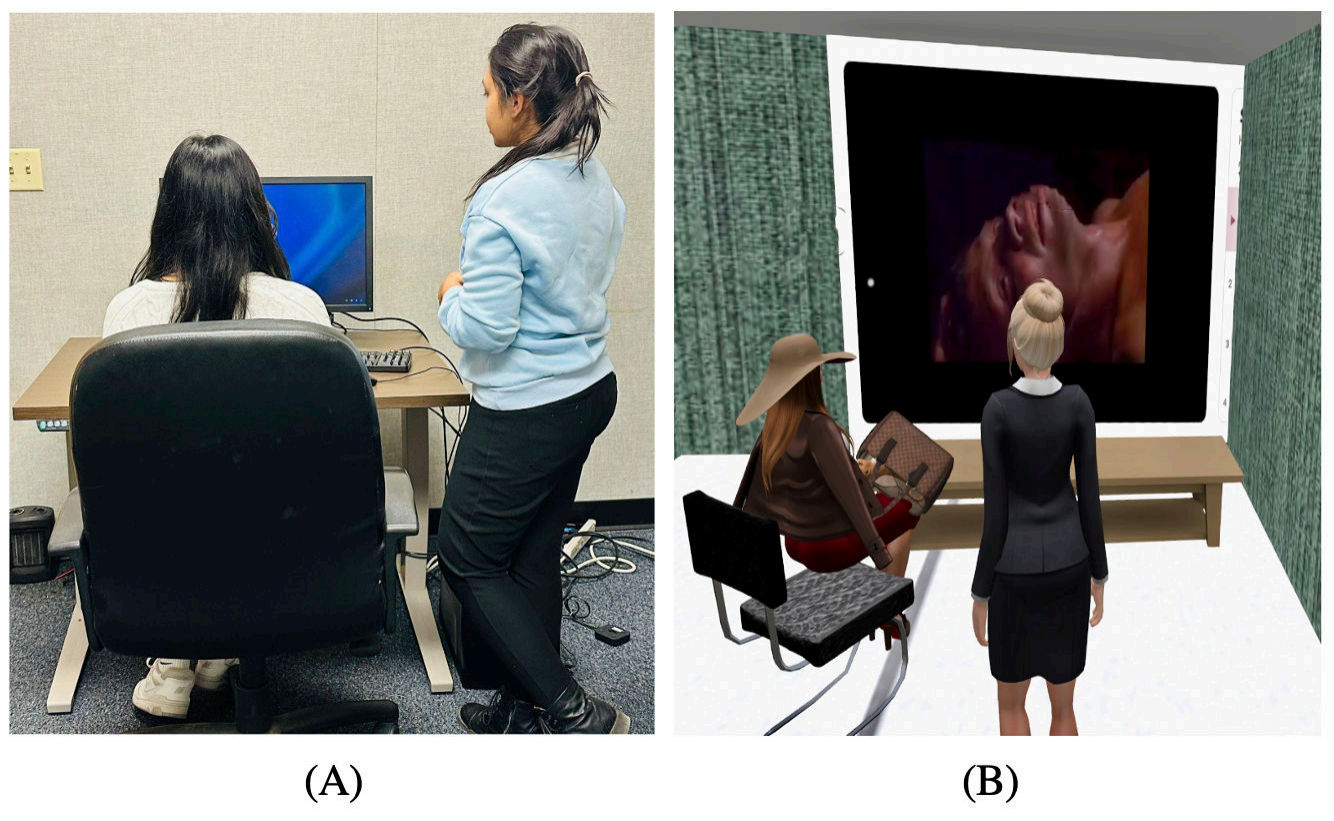
Experimental settings used in the study: (A) real-world laboratory environment, consisting of a soundproof room equipped with a desktop computer, table, and chair, where participants viewed the video stimuli on YouTube, and (B) Metaverse environment created on the Second Life platform, consisting of a virtual room modeled after a real-world setting and equipped with a projector, table, and chair, where participants viewed video stimuli via avatars.

The real-world setting for this study consisted of a soundproof laboratory room located at Binghamton University. The room was furnished with a desktop computer, a table, and a chair. Participants were invited to the lab to watch the videos on the desktop using YouTube as the media platform. The layout of the real-world setting is shown in Figure 1A.

### Metaverse Settings

For the Metaverse environment, we used the SL platform. A room replicating the real-world setting was created within the SL environment. This virtual room was equipped with a projector for video playback, as well as a table and a chair. Two customizable avatars were developed: One represented the researcher, and the other represented the participant. A short training video was provided to the participants beforehand to familiarize them with the SL system. This training video included instructions on navigation, communication through talking and chatting, and watching videos within the SL environment. The setup of the SL environment is shown in Figure 1B.

### Emotional-Eliciting Task

In this experiment, we asked the participants to watch short emotional clips as an emotional-eliciting task. We selected 5 short videos to elicit emotions including amusement, anger, disgust, fear, and sadness. These emotions were chosen for their relevance to therapeutic applications for depression. The video clips were identified through a literature review of previous studies that successfully used these stimuli to evoke the targeted emotions. Table 1 describes each video clip and its associated emotion.

**Table 1.** Description of emotional-eliciting video stimuli used in the experiment, summarizing the targeted emotion, film name, scene content used to provoke the intended emotional response, clip duration, and supporting reference.

Targeted emotion	Film name	Description of clip	Length (seconds)	Reference
Amusement	I Love Lucy	Clip of a candy factory where two women struggle with the rapid speed of a conveyor belt and start stuffing the chocolate candies into their mouths	178	[45]
Anger	My Bodyguard	Clip of a moment where two bullies attack a young boy, beat him, break his bike, and throw it into the water	249	[46]
Disgust	Pink Flamingos	Clip of a scene where a person eats dog feces	29	[46]
Fear	Silence of the Lambs	Clip of a scene where an FBI agent pursues a suspect into the basement and uncovers a decomposing corpse	216	[46]
Sadness	The Champ	Clip of a moment where a young boy tearfully calls out to a boxer, who, severely injured after a match, collapses in the locker room and takes his final breaths, leaving those around him devastated	158	[46]

### Participant Recruitment

After receiving approval from the Institutional Review Board of Binghamton University, a brief informational leaflet about the study and contact information for the primary investigator and researcher were distributed across Binghamton University’s physical and virtual locations. Inclusion criteria required the participants to be adults aged 18 years to 35 years, with or without prior experience with virtual environments. Exclusion criteria included uncorrectable visual impairments that prevented individuals from achieving normal or functional vision as well as severe motion sickness or a known intolerance to virtual environments. The researcher initially screened the interested individuals based on inclusion and exclusion criteria, and participants who met the inclusion criteria were invited to visit the Human Cognitive and Visual Behavior Lab at Binghamton University. We recruited 14 healthy individuals (with either no or minimal symptoms of depression), as well as 14 individuals with self-reported depressive symptoms.

### Experimental Procedure

In the real-world setting, the experimental procedure was described upon the participants’ arrival at the laboratory, and informed consent was obtained. Next, they were asked to complete the PHQ-9 questionnaire and provide demographic information, including age and gender. Participants were then instructed to complete the emotional elicitation task, which involved watching 5 emotional video clips on YouTube using a desktop computer. In this setting, communication between the researcher and participants occurred face to face.

For the Metaverse group, participants received a prerecorded training video with instructions on navigating SL, the Metaverse platform used in this study, and an electronic copy of the informed consent form. They were asked to review these materials, submit the signed informed consent, and reach out with any questions for clarification before attending their scheduled session. The SL environment was preconfigured on the laboratory’s desktop computer, including a virtual room with a video playback projector. Upon arrival, participants were guided through the procedure virtually, with all communication between the researcher and participants conducted through SL avatars to eliminate direct interaction. After completing the PHQ-9 questionnaire and providing their demographic data, participants performed the emotional eliciting task of watching the 5 emotional video clips using the SL platform.

Participants from both groups completed the 16-item self-report inventory [46] to report their emotional arousal after watching each video clip. To reduce fatigue, a 2-minute rest period was provided between clips. The video clips were presented randomly for each participant to control for order effects. After viewing all 5 video clips and completing the associated self-report inventories, participants completed the NASA-Task Load Index (NASA-TLX) [47] and Positive and Negative Affect Schedule (PANAS) [48] questionnaires to report their perceived MWL and emotional engagement, respectively. Upon completing the experiment, each participant received a US $15 Amazon gift card for their time and participation.

### Outcome Measures

#### Patient Health Questionnaire-9

The PHQ-9 [44], a brief self-report questionnaire, was used to screen for depression and its severity among participants. This 9-item measure includes 8 items addressing depressive symptoms and 1 item evaluating suicidal ideation, each corresponding to the diagnostic criteria for major depressive disorder as outlined in the *Diagnostic and Statistical Manual of Mental Disorders, Fourth Edition* (*DSM-IV)*. Participants were asked to rate how frequently they experienced these symptoms over the past 2 weeks using a 4-point scale ranging from 0 (not at all) to 3 (nearly every day). The final PHQ-9 score, ranging from 0 to 27, indicates depression severity, with established thresholds used to categorize the level of depressive symptoms. Scores of 0 through 4 indicate minimal or no depression, 5 through 9 reflect mild depression, 10 through 14 indicate moderate depression, 15 through 19 represent moderately severe depression, and 20 through 27 signify severe depression. Additionally, the PHQ-9 includes an item that assesses functional impairment by asking participants who reported any symptoms, “How difficult have these problems made it for you to do your work, take care of things at home, or get along with other people?” [49]. For this study, participants scoring between 0 and 4 on the PHQ-9 were classified as healthy participants with minimal or no depressive symptoms and placed in the minimal or no depression group. In contrast, those scoring 5 or higher were categorized as experiencing self-reported depressive symptoms and placed in the self-reported depressive symptoms group.

#### 16-Item Self-Report Inventory

This study used a 16-item self-report inventory to assess discrete emotions. The items included amusement, anger, arousal, confusion, contempt, contentment, disgust, embarrassment, fear, happiness, interest, pain, relief, sadness, surprise, and tension. Participants were asked to rate each item on a 9-point scale, ranging from 0 to 8, where 0 indicated no experience of the emotion and 8 represented the highest intensity of the emotion they felt. The order of the 16 items was randomized for each participant. This rating procedure was adapted from the study by Gross and Levenson [46].

#### NASA Task Load Index

The NASA-TLX [47], a multidimensional, survey-based tool, was used to collect subjective feedback on MWL following the emotional-eliciting task. The NASA-TLX includes 6 subscales: mental demand (MD), physical demand (PD), temporal demand (TD), frustration, effort, and performance. To assess MWL, participants rated each of the 6 subscales on 20-step bipolar scales based on their experience after completing the task. Each scale generated a score of 0 to 100, rounded to the nearest multiple of 5. To determine the weight of each subscale in overall MWL, participants completed 15 paired comparisons, representing all possible permutations of the 6 subscales. The overall MWL score was calculated as the weighted mean of the 6 dimensions, yielding a score between 0 and 100, where low scores indicate low MWL and high scores indicate high MWL [50].

#### Positive and Negative Affect Schedule

In this experiment, we used the PANAS [48], a widely used adjective-based questionnaire, to assess the participants’ emotional engagement. The PANAS consists of 10 positive affect (PA) terms and 10 negative affect (NA) terms, which measure pleasurable feelings and distress or unpleasurable feelings toward a situation, respectively. The specific PA and NA terms used in the scale are listed in Textbox 1. To assess participants’ emotional responses, they were asked to rate how much they experienced each of the 20 emotions after completing the task, using a Likert scale ranging from 1 to 5. Lower scores on the Likert scale indicated a weaker association with the specific term, while higher scores indicated a stronger association. The final PA and NA scores were calculated by summing the 10 positive and 10 negative terms, respectively [51].

Textbox 1. **Positive affect (PA) and negative affect (NA) terms from the Positive and Negative Affect Schedule (PANAS) scale used to assess emotional responses.**

PA:

- Attentive
- Active
- Alert
- Excited
- Enthusiastic
- Determined
- Inspired
- Proud
- Interested
- Strong

NA:

- Hostile
- Irritable
- Ashamed
- Guilty
- Distressed
- Upset
- Scared
- Afraid
- Jittery
- Nervous

### Data Analysis

#### Sample Size Calculation

An a priori power analysis was conducted using G*Power software to determine the required sample size. The effect size was estimated from pilot data (the means and SDs of the NASA-TLX scores) involving 5 participants in each group. Using a 1-tailed test with *α*=.05 and *β*=0.20, the required sample size was calculated as 5 participants in each group. To improve data accuracy, we included 14 participants in each group.

#### Statistical Analysis

We collected 4 sets of subjective data in this experiment: PHQ-9, 16-item self-report inventory, NASA-TLX, and PANAS. The PHQ-9 total score was used to classify participants into 2 categories based on depression severity: minimal or no depression and self-reported depression symptoms. For the 16-item self-report inventory, scores from 5 dimensions corresponding to the 5 video clips were analyzed (Table 2). All 6 subscales of the NASA-TLX, including MD, PD, TD, performance, effort, frustration, and the overall MWL, were evaluated. PANAS scores for PA and NA were calculated by summing each dimension’s emotional factors. Table 2 lists the dependent and independent variables for this study. Data analysis was performed using SPSS version 28 (IBM Corp). To examine the effect of the independent variables on the dependent variables, a 2-way ANOVA was conducted. The threshold for statistical significance was set at *P*<.05. To compare the means for any significant main effect or interaction effect, we used the Tukey least significant difference (LSD) method.

**Table 2.** List of independent and dependent variables in the experiment.

Variables	Items measured
Independent variables	
Environment	Real world Metaverse
Depression severity	Minimal or no depression Self-reported depression
Dependent variables	
16-item self-report inventory	Amusement Anger Disgust Fear Sadness
NASA-TLX^a^	Mental demand Physical demand Temporal demand Performance Effort Frustration Overall MWL^b^
PANAS^c^	Positive affect Negative affect

a NASA-TLX: NASA Task Load Index.

b MWL: mental workload.

c PANAS: Positive and Negative Affect Schedule.

### Ethical Considerations

This study was reviewed and approved by the Institutional Review Board of Binghamton University (IRB ID: STUDY0000402; date of approval: July 19, 2023). All participants provided informed consent before participation. All collected data were de-identified before analysis. All collected data were stored on a password-protected computer, with no personally identifiable information. All participants received a US $15 Amazon gift card as a compliment upon completing their study session.

## Results

### Participants

We recruited 28 individuals, evenly divided into 2 groups based on their depression severity: minimal or no depression and self-reported depression. Participants were assigned to either the real-world or SL environment, with each condition including 7 participants from both depression severity groups. Demographic information for all participants is presented in Table 3.

**Table 3.** Demographic characteristics of participants across the real-world and Second Life (SL) environment groups.

Environment		Depression level					
		Minimal or no depression		Self-reported depression		Total sample	
		Participants, n	Age (years), mean (SD)	Participants, n	Age (years), mean (SD)	Participants, n	Age (years), mean (SD)
Real world							
Gender							
Male		5	23.60 (5.68)	4	25.25 (1.50)	9	24.33 (4.21)
Female		2	20.50 (0.71)	3	23.00 (4.36)	5	22.00 (3.39)
Total sample		7	22.72 (4.89)	7	24.29 (2.98)	14	23.50 (3.98)
SL							
Gender							
Male		4	22.75 (4.79)	3	25.33 (2.08)	7	23.86 (3.85)
Female		3	27.33 (0.58)	4	22.75 (5.62)	7	24.72 (4.68)
Total sample		7	24.72 (4.19)	7	23.86 (4.38)	14	24.29 (4.14)

### 16-Item Self-Report Inventory

**Figure 2.**
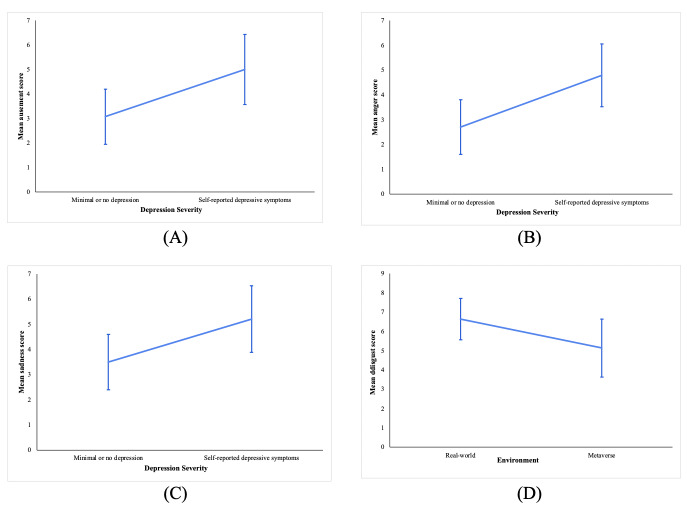
Effects of depression severity and environment on dependent variables showing mean scores and 95% CIs: (A) main effect of depression severity on mean amusement score, (B) main effect of depression severity on mean anger score, (C) main effect of depression severity on mean sadness score, and (D) main effect of environment on mean disgust score.

The 2-way between-subjects ANOVA showed that depression severity had a significant effect on the mean amusement scores reported by the participants (*F*_1,24_=4.882, *P*=.04). Participants with minimal or no depression (mean 3.07, SD 1.940) reported a significantly lower mean amusement score than the participants with self-reported depression symptoms (self-reported depression: mean 5.00, SD 2.481; Figure 2A).

The 2-way ANOVA showed that depression severity had a significant effect on the mean anger scores reported by the participants (*F*_1,24_=7.067, *P*=.01). Participants with minimal or no depression (mean 2.71, SD 1.899) reported a significantly lower mean anger score than participants with self-reported depression symptoms (mean 4.79, SD 2.190; Figure 2B).

The 2-way ANOVA showed that depression severity had a significant effect on the mean sadness scores reported by the participants (*F*=4.353, *P*=.048). Participants with minimal or no depression (mean 3.50, SD 1.912) reported a significantly lower mean sadness score than participants with self-reported depression symptoms (mean 5.21, SD 2.293; Figure 2C).

The 2-way ANOVA revealed a trend suggesting that the environment may influence the mean disgust scores reported by the participants (*F*=3.128, *P*=.09). Participants who watched the video clips in a real-world environment (mean 6.64, SD 1.865) reported a higher mean disgust score than those who watched the video clips in a Metaverse environment (mean 5.14, SD 2.598; Figure 2D).

### NASA Task Load Index

**Figure 3.**
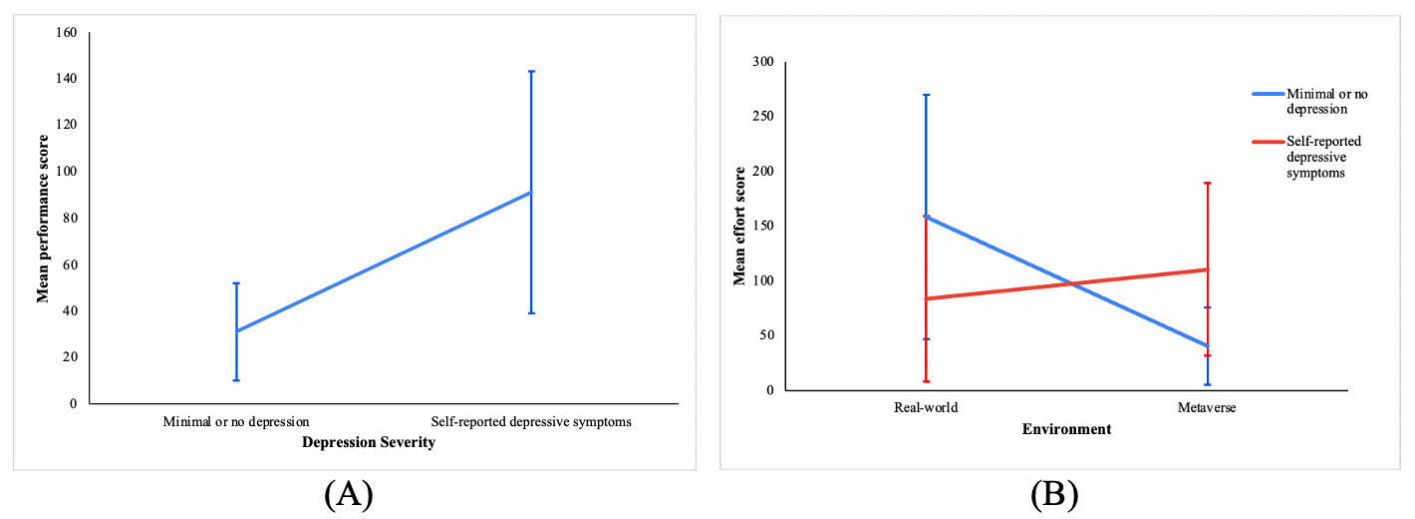
Effects of depression severity and environment on dependent variables showing mean scores and 95% CIs: (A) main effect of depression severity on mean performance score and (B) interaction effect of environment and depression severity on mean effort score.

The 2-way ANOVA showed that depression severity had a significant effect on the mean performance scores reported by the participants (*F*=4.939, *P*=.04). Participants with minimal or no depression (mean 31.07, SD 36.278) reported a significantly lower mean performance score than participants with self-reported depression (mean 91.07, SD 90.302; Figure 3A).

The 2-way ANOVA showed that depression severity and environment had no significant main effect on the mean effort perceived by the participants; however, a significant interaction effect was discovered (*F*_1,24_=4.931, *P*=.04). The Tukey LSD revealed that there was a significant difference between the mean effort score perceived by the participants with minimal or no depression in the 2 different environments, where participants in the real-world environment reported higher effort scores than those in the Metaverse environment (real world: mean 158.571, SD 120.441; *P*=.02; Figure 3B).

### Positive and Negative Affect Schedule

The 2-way ANOVA showed that neither the environment nor the severity of depression significantly affected the mean PA and NA scores reported by the participants.

## Discussion

### Principal Findings

This study aimed to evaluate the potential of a Metaverse environment for eliciting therapeutic emotions compared with a real-world setting. Consistent with our aim, we compared individuals’ emotional arousal, perception of MWL, and emotional engagement while performing an emotional elicitation task in the Metaverse and in a real-world setting. We recruited 28 participants, both with minimal or no depression symptoms and with self-reported depression symptoms, to perform the emotional-eliciting task in the Metaverse and real-world environments using the SL platform and a desktop computer, respectively. Subsequently, they rated their perceptions of emotional arousal, MWL, and emotional engagement immediately after completing the tasks. We found that depression severity significantly influenced emotional arousal, with individuals reporting depressive symptoms demonstrating higher levels of amusement, anger, and sadness across both environments. There was a nonsignificant trend suggesting higher disgust ratings in the real-world condition than in the Metaverse. We also observed a significant interaction between depression severity and environment on the perceived effort score, with participants with minimal or no depression reporting higher effort in the real-world environment than in the Metaverse. Additionally, a significant effect of depression severity was observed on the mean performance score, where participants with self-reported symptoms perceived higher performance than those with minimal or no depression. No significant differences were found between the environments or depression severity in overall MWL, MD, PD, TD, frustration, or emotional engagement, including PA and NA.

### Interpretation and Implications

The elevated emotional responses among participants with self-reported depressive symptoms aligned with well-established evidence that individuals with depression often exhibit hyperarousal and intensified emotional reactivity, characterized by prolonged and heightened responses to emotional cues [37,38]. This finding is consistent with prior research indicating that individuals with depressive symptoms often experience heightened emotional arousal in response to stimuli [36,38]. Interestingly, disgust responses were slightly higher in the real-world environment than in the SL environment, which may align with prior evidence indicating that highly realistic or naturalistic stimuli tend to elicit stronger aversive responses, particularly for negatively valenced emotions such as disgust [41]. This result highlights that Metaverse environments may attenuate negative emotional arousal compared with real-world settings, suggesting a potential advantage in reducing such affective responses in ways that might be beneficial within therapeutic contexts, although such implications should be considered preliminary. However, the study did not find significant differences in emotional arousal (amusement, anger, sadness, and fear) as a function of environment. In therapeutic contexts, this pattern suggests that, although the Metaverse may help reduce certain aversive reactions, it may tentatively maintain typical emotional engagement, supporting its potential feasibility as an alternative therapeutic platform for future investigation.

An interesting finding of this study was the significant interaction effect of environment and depression severity on perceived effort. Participants with minimal or no depressive symptoms reported greater effort in the real world than in the Metaverse, suggesting that the Metaverse environment required less effort to achieve similar performance levels. Additionally, the absence of any significant differences in overall MWL, MD, PD, TD, or frustration between the real-world and Metaverse environments, as well as between the 2 groups, tentatively suggests that individuals with self-reported depression symptoms may not perceive these MWL dimensions differently from those without symptoms. One possible explanation is the high degree of task similarity between the two environments, as the Metaverse setting was intentionally designed to closely replicate the real-world tasks, potentially minimizing perceived differences. Another contributing factor may be the relatively brief exposure to each environment, combined with rest periods between sessions, which could have attenuated differences in workload perception. Interestingly, in our study, depression severity was significantly associated with higher perceived performance, which reflects a heightened perception of being successful in accomplishing the task among individuals with self-reported depression symptoms. In contrast to these findings, prior research indicates that depression is often associated with workload and impaired performance, which is aggravated by exposure to cognitive stressors such as high mental demands [42,43,52]. These findings suggest that, if designed carefully, the virtual environment of the Metaverse may potentially provide a comparable experience to the real world in terms of workload perception, even among individuals with varying mental health conditions.

Emotional engagement did not differ significantly between the Metaverse and real-world settings, suggesting that the environments may elicit comparable positive and negative responses. Although prior studies reported mixed emotional experiences in virtual environments [15], the Metaverse may help alleviate negative emotions such as fear or boredom by providing opportunities for exploration and fostering positive affective states like contentment, curiosity, and enthusiasm [15,53]. At the same time, extended engagement in virtual spaces may contribute to social alienation, with negative emotions potentially affecting real-life interactions and exacerbating isolation, anxiety, or identity-related challenges [18,20]. Additionally, interactions mediated through avatars cannot fully replicate tactile emotional expressions, such as handshakes or hugs, which may limit the depth of emotional engagement [18]. Future research should investigate specific interventions or design features to enhance emotional involvement and support well-being in Metaverse environments.

The Metaverse shows promise as a platform for eliciting emotional responses relevant to therapeutic contexts, supporting emotional arousal and engagement that may be comparable to real-world experiences. Its interactive nature may also reduce mental demands, providing a less effortful environment for individuals who may face challenges in traditional settings. These features suggest that Metaverse environments could potentially be leveraged to simulate real-life emotional situations in a controlled, adaptable, and accessible manner, aligning with the goal of facilitating emotional processing in therapeutic applications. Future research should explore personalized virtual interventions, guided emotional regulation exercises, and immersive exposure strategies to further enhance the potential of Metaverse-based experiences in supporting mental health.

### Limitations

Several limitations of this study should be acknowledged. First, the study sample was restricted to adults aged 18 years to 35 years, which limits the generalizability of the findings to other age groups. Future studies should include a more diverse range of participants to determine whether the observed effects extend across different ages. Second, the study relied exclusively on self-reported measures of emotional arousal, MWL, and emotional engagement. Although these instruments provide subjective insights, they inherently limit the objectivity of the findings and may be influenced by individual biases. Incorporating complementary physiological measures, such as galvanic skin response and heart rate variability, alongside clinical data to detect depression could provide a more comprehensive and robust evaluation. Third, this study was a preliminary investigation with a small sample size: 7 participants per group in a 2×2 factorial design. The limited statistical power increases the likelihood of Type II errors, meaning that nonsignificant results cannot be interpreted as evidence of equivalence between the Metaverse and real-world conditions. As such, the findings should be viewed as initial, exploratory insights intended to inform future, larger-scale research rather than definitive conclusions. Fourth, the participants in the experiment were exposed to the real world and the Metaverse environment for a brief period. Prolonged exposure may reveal additional insights and more significant findings. Finally, the study utilized a single Metaverse platform, the SL. As an older, nonimmersive platform with dated graphics, SL may not fully capture the capabilities of immersive extended-reality systems. This limitation affects ecological validity, and findings may not generalize to other contemporary Metaverse platforms with higher levels of interactivity, realism, and immersion. Future research should examine multiple, advanced Metaverse platforms with varying levels of immersion or interactivity to evaluate their potential for therapeutic applications.

### Conclusions

This pilot study provides early insight into how a screen-based Metaverse environment, Second Life, may support emotional elicitation tasks commonly used in therapeutic contexts. Although the findings suggest that participants reported tentatively comparable levels of emotional arousal, perceived MWL, and emotional engagement across the Metaverse and real-world settings, these results should be interpreted cautiously given the small sample size; reliance on self-report measures; and use of a nonimmersive, older virtual platform. This work is an initial step toward exploring the potential of Metaverse environments for future mental health research, such as helping to reduce emotional distress, provide controlled exposure, and alleviate cognitive stress, particularly for individuals who may find real-world environments overwhelming. It highlights the need for future studies with larger clinical samples and more ecologically valid therapeutic tasks.

## Abbreviations

AI: artificial intelligence
DSM-IV: Diagnostic and Statistical Manual of Mental Disorders, Fourth Edition
LSD: least significant difference
MWL: mental workload
NA: negative affect
NASA-TLX: NASA Task Load Index
PA: positive affect
PANAS: Positive and Negative Affect Schedule
PD: physical demand
SL: Second Life
TD: temporal demand
WHO: World Health Organization
